# Large language models rewrite the diagnostic rheumatology playbook

**DOI:** 10.1016/j.ero.2025.09.002

**Published:** 2025-10-10

**Authors:** Diego Benavent, Montserrat Roig-Kim, Marti Aguilar-Coll, Joan M Nolla

**Affiliations:** Department of Rheumatology, IDIBELL-Hospital Universitari de Bellvitge, University of Barcelona, Barcelona, Spain

In this issue of the journal, Kremer et al present a timely head-to-head evaluation of 4 general-purpose large language models (LLMs) and 3 widely used diagnostic decision-support systems (DDSSs) for the identification of rare rheumatic diseases. Using 60 carefully curated vignettes, of which 10 were unpublished, the investigators report that LLMs proposed the exact diagnosis in 35% of cases, 4-fold the performance of DDSSs (8.9%; *P* < .001). Mean diagnostic scores likewise favoured LLMs (3.5 vs 2.1), and the median time to a result fell from >3 minutes to roughly 20 seconds. This evaluation showed how general-purpose LLMs can outperform established DDSS comparators on the assessment of certain rheumatic diseases. A key methodological strength is the inclusion of both published and unpublished clinical vignettes, which improves the external validity of the results while reducing the likelihood that LLMs would merely reproduce training data. In addition, the authors included a broad spectrum of rare diseases, as well as a subset of differential diagnoses, thus suggesting that LLMs may be helpful regardless of disease category.

We are encouraged by the reported improvements in diagnostic accuracy and speed of LLMs compared with legacy DDSSs. LLMs are characterised by an ease of use that allows for a wide implementation throughout the medical setting. In fact, the authors found that a simple prompt is sufficient to obtain good diagnostic support from the model, whereas the use of complex prompts did not provide any decisive added value. The potential to expedite complex diagnostic pathways and refine medical training is evident, and this new cognitive workflow shows potential to shape how we conceive medical practice and learning nowadays. Given the inevitability of LLMs in clinical environments, undergraduate curricula must evolve to ensure that future physicians develop the capacity to critically appraise artificial intelligence (AI)-generated output, identify errors, and remain accountable for clinical decisions. In this regard, the use of LLM must be matched by thoughtful consideration of clinical utility and safety. The same fluency that facilitates diagnosis can mask confident errors, a phenomenon well recognised as ‘hallucination’. Hallucinations are especially hazardous in rare disease diagnostics as well as complex decision making, where confirmatory literature may be sparse and the clinical stakes high. Rigorous oversight, therefore, remains essential; LLM-generated suggestions should augment, not replace, clinician judgement.

The study raises important questions about ethical and regulatory challenges in using LLMs for clinical decision support. If these tools influence direct patient care, it remains uncertain whether they should be regulated as medical devices. There is a growing debate that algorithms designed to provide advice on diagnosis or management may require appropriate oversight to ensure their safety and effectiveness. Clear regulatory frameworks could mitigate the risk of premature use and safeguard patient interests. Achieving these aims calls for transparent reporting of performance metrics, robust validation studies, including collaborations among clinicians, researchers, industry partners, and policymakers. Looking ahead, professional societies may consider the safe use of AI-based decision support as part of centre-of-excellence and quality-assurance criteria in rheumatology. Any such requirements should be dependent on demonstrated clinical benefit and should prioritise process standards (such as incident reporting or human oversight) rather than requiring specific vendors or models.

Beyond diagnostic tasks, these models can serve as dynamic engines for medical education, allowing medical students, trainees, and practitioners to interrogate complex clinical scenarios in near real time while progressively uncovering layers of information. Mastery of clinical reasoning begins with understanding the nuances of disease presentation and the practice of differential diagnosis. As LLMs automate this task with increasing fluency, educators must consider how their use may inadvertently displace rather than reinforce critical thinking. A trainee faced with an atypical pattern of osteitis can ask follow-up questions, request stepwise disclosure of laboratory or imaging data, and challenge the model to justify each branch of its differential diagnosis, thereby transforming a static vignette into an interactive exchange. Because LLMs can also generate plausible alternative presentations, they lend themselves to deliberate‐practice drills in which learners must sift through distracting findings and select the most judicious investigations. Moreover, the models’ ability to cite primary literature and highlight pathophysiological mechanisms on demand promotes evidence-based reasoning and helps bridge the gap between textbook knowledge and bedside decision making. Indeed, modern LLMs can generate step-by-step rationales, known as ‘chain-of-thought’, that resemble elements of clinical reasoning. Used judiciously in supervised educational settings, such rationales can support hypothesis generation, selection of next tests, and reflection on why alternatives were rejected. In clinical use cases, LLM-based rationales should be treated as exploratory rather than evidence, and interfaces should avoid presenting unverifiable reasoning as fact; concise, reference-linked justifications or structured evidence traces offer a safer compromise. In this regard, to safeguard educational integrity, LLMs should be deployed within a pedagogical framework that encourages critical reflection and hypothesis refinement, not shortcutting the diagnostic process. When integrated into structured curricula with supervisory checkpoints, these features can foster reflective learning, accelerate pattern recognition, and, ultimately, cultivate more resilient clinical judgement without sacrificing patient safety.

The authors also touch upon a pivotal issue rarely addressed in medical studies: deployment architecture. An open-source, locally hosted model (Llama 3.3) ranked third overall and outperformed traditional DDSSs, hinting at a future in which hospitals can run privacy-preserving models behind their own firewalls. Such configurations may mitigate concerns about proprietary data transfer and enable fine-tuning on institution-specific cohorts. When clinical text is sent to cloud-hosted, general-purpose LLMs, that transmission constitutes processing of personal data and protected health information; institutions therefore require explicit data-processing agreements, documented retention, and robust deidentification. Local deployment can reduce data-exfiltration risk but is associated with capital and operating costs that many providers may find prohibitive. Pragmatic hybrid approaches, such as services that filter and pseudonymise prompts, can balance privacy, performance, and feasibility. Whether these systems fall under the horizon of medical-device regulation will depend less on model weights than on the claims made and the degree of clinical autonomy conferred. Policymakers will need to balance the agility of open ecosystems with the safeguards traditionally applied to diagnostic software.

Methodologically, the study is notable for transparent scoring rules and the use of identical prompts across platforms. Nonetheless, there are some aspects that may deserve further research in future studies. Firstly, performance with structured vignettes may overestimate real-world utility, where data are heterogeneous and sometimes contradictory. Secondly, latency was measured in trigger-to-output time; total consultation time, including prompt crafting and clinician verification, was not captured. Thirdly, numerical differences in effectiveness were observed between disease categories, but it is unknown whether LLMs are more helpful in some particular subsets because no comparisons were made. Finally, equity considerations, including how models perform across age, sex, and ethnically diverse presentations, remain unexplored.

Kremer et al provide compelling evidence that LLMs have crossed a threshold in both speed and diagnostic yield for rare rheumatic diseases. Prospective studies assessing educational and clinical workflows will reveal whether these early gains translate into earlier diagnoses and better outcomes; rheumatologists should therefore engage proactively with developers and regulators to improve model evolution towards transparent and clinically relevant solutions.

## Competing interests

DB is part time employed by Savana. The other authors declare that they have no known competing financial interests or personal relationships that could have appeared to influence the work reported in this paper.

